# Statistical analysis of Nkoulou soils properties and suitability for earthen constructions

**DOI:** 10.1016/j.heliyon.2022.e11141

**Published:** 2022-10-19

**Authors:** Tchedele Langollo Yannick, Taïga Oumar Ali, Darman Japhet Taypondou, Michel Ivan Leke Fekwe Kom, Arnold Liyong Luc, Luc Leroy Mambou Ngueyep, Jacques Richard Mache

**Affiliations:** a Research Department, Local Materials Promotion Authority (MIPROMALO), Yaounde, Cameroon; b Department of Mining Engineering, School of Geology and Mining Engineering, University of Ngaoundere, Meiganga, Cameroon; c Laboratory of Clays, Geochemistry and Sedimentary Environments (AGEs), Boulevard du Rectorat, 17 (Bât. B18) Sart Tilman – 4000, Liege, Belgium

**Keywords:** Compressed earth brick, Stabilization, Road construction, Statistical correlations, Mathematical models

## Abstract

The present work consists of sampling, characterizing, classifying, and studying the soils of the locality of Nkoulou for the manufacture of cement-stabilized compressed earth bricks (CEBs) and possible use in road building. 08 samples were taken on 02 sites. Chemical and mineralogical characterization identified these soils as ferric-dominated laterites consisting of quartz, illite, hematite, kaolinite, goethite, gibbsite, muscovite, and magnesite associated with trace minerals. The studied soils have an average natural water content of 11.73% and average values of specific gravity of 2.51 and 2.48 (respectively for site N°1 and site N°2). These soils are mainly composed of gravel followed by sand and are classified as Group B fine-grained soils according to the French Road Grading Manual (GTR). The natural water content (W) shows a good correlation with clay content (R = 0.75), and silt content (R = 0.73). The CBR Index (ICBR) meanwhile has a good correlation with the silt content (R = −0.69), similar between ICBR and maximum dry density (R = 0.73). According to CRATERRE Nkoulou soils can be used as well in the manufacture of CEBs as in the realization of road works in layers of fill. The cement stabilization of the compressed soil specimens allows the mechanical strength to increase with the stabilization rate, just as the water absorption of the specimens which also varies with the fines content, and the curing time according to the correlation study. These results allow us to establish the predictive model of compressive strength and flexural strength as a function of cement content and time with R^2^ = 0.75 and R^2^ = 0.93, respectively.

## 1 Introduction

Developing countries are facing a real deficit in decent housing, despite a potential in raw materials sometimes identified. Resolving this deficit depends on the quality and availability of construction materials. Among the latter, lateritic soils have shown good applications in construction (Kerali, 2001; Guettala et al., 2002; Millogo et al., 2008; Kwon et al., 2010) and are present at about 33% in the intertropical zone (Tardy, 1992). Cameroon is essentially found in the intertropical zone (Pedro, 1968), which induces an abundant presence of lateritic soils.

In addition, the use of lateritic soils for BTC requires very little energy. Moreover, the refractory properties of earth bricks allow for a reduction in the use of heating and cooling inside buildings (Allinsonet Hall, 2010). These characteristics indicate a real advantage from the environmental point of view compared to the traditional blocs in micro-concretes whose use is more widespread in the building sites.

In this context favorable to the valorization of lateritic soils, many works have been carried out in the direction of the study of laterites for the production of CEB and clay bricks, but also as a foundation layer for various civil engineering works such as roads and associated engineering structures (Kassogue et al., 2002; Keyangue Tchouata et al., 2019). The geotechnical behavior of lateritic soils depends on several parameters (Ngon ngon et al., 2012; Nzeukou Nzeugang et al., 2021) showed that particle size, plasticity, chemical and mineralogical composition influenced the geotechnical behavior of soils. Wu et al. (2017) established among others that soil binding agents interacted significantly with pore characteristics. Other authors have shown that the addition of stabilizers variably influenced the mechanical properties of soils (Akbarimehr et al., 2021; Lemaire et al., 2013; Mengue et al., 2017; Kagonbé et al., 2020). For Akbarimehr et al. (2021) the addition of rubber waste allowed to change the types of failure of pure Tehran clay to a ductile behavior, while Lemaire et al. (2013) showed that the combined addition of lime + cement had the effect of causing the formation of C-(A)-S-H which improves the mechanical properties, during the curing period. Regarding (Mengue et al., 2017; Kagonbé et al., 2020), their work showed the positive impact of the addition of cement as a stabilizer for CEBs, especially on the mechanical resistance. All of these works having been carried out with the aim of realizing works respecting defined geotechnical criteria. In practice, the realization of the tests sometimes comes up against financial constraints. To overcome these constraints, some authors have proposed to establish prediction models of soil behavior (Mola-Abasi and Eslami, 2019; Naeini et al., 2018; Hassanlourad et al., 2017). Thus, Mola-Abasi and Eslami (2019) used the Group Method of Data Handling (GMDH)-type Neural Network (NN) and Genetic Algorithms (GAs) to predict the Cohesion (C) and friction angle (φ) of marine soils. Results has shown that the polynomial models are appropriate models to estimate shear strength parameters (C and φ). Naeini et al. (2018) studied the suitability of the Group Method of Data Handling (GMDH) polynomial Neural Network (NN) to estimate the subgrade reaction modulus (Ks) of clayey soils, resulting in an improvement in predicting Ks. The work of Hassanlourad et al. (2017) concerned the estimation of the compaction parameters (γ_dmax_ and ω_opt_) using Group Method of Data Handling (GMDH) type neural network (NN) with successful results.

In the same vein, this work proposes to make a study of the soils of the locality of Nkoulouu in the district of Nkol-Afamba, because these soils are used in many works, despite a lack of information on their properties. The objective is to identify and characterize the soils of the area for the manufacture of cement-stabilized compressed earth bricks (CEBs) and their classification for possible use in road construction. In a specific way, it will be a question of carrying out a sampling campaign. Thereafter, the laboratory work will consist in determining the physicochemical and mineralogical parameters of the studied soils, making specimens of compressed and stabilized earth bricks, and studying their mechanical behaviors. Finally, the acquired data will allow classifying these soils, realizing the correlation studies, and bringing out models of prediction of the mechanical strengths according to the most correlated parameters.

## 2 Geographical context

**Figure 1:**
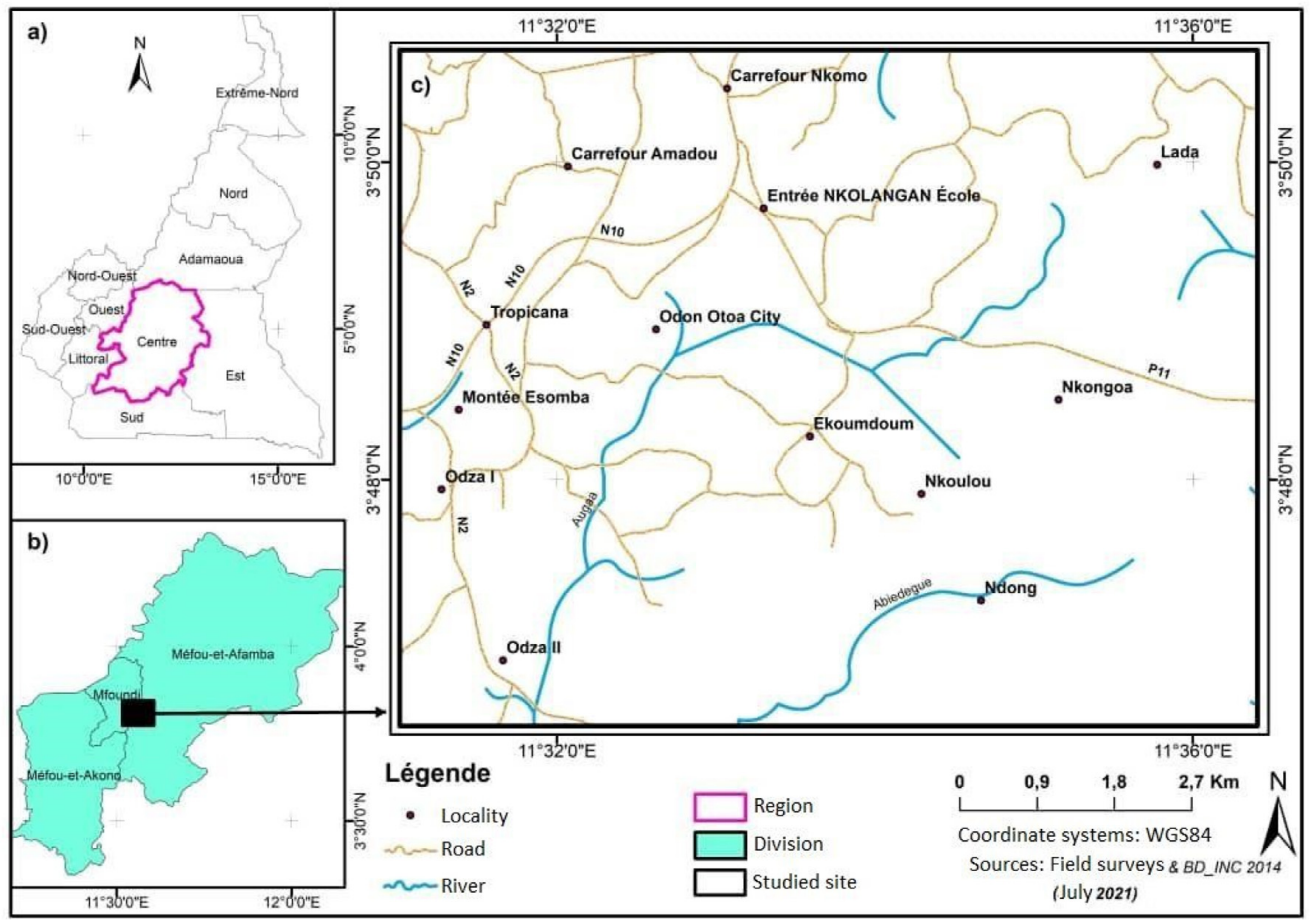
Location map of the study area.

The study area is located in the locality of Nkoulou, in the southwestern part of the Mefou et Afamba Division, Nkol-Afamba Subdivision, in the Central Cameroon Region. Figure 1 illustrates the geographic context of the study area.

## 3 Sampling campaign and description of soil profiles

**Figure 2:**
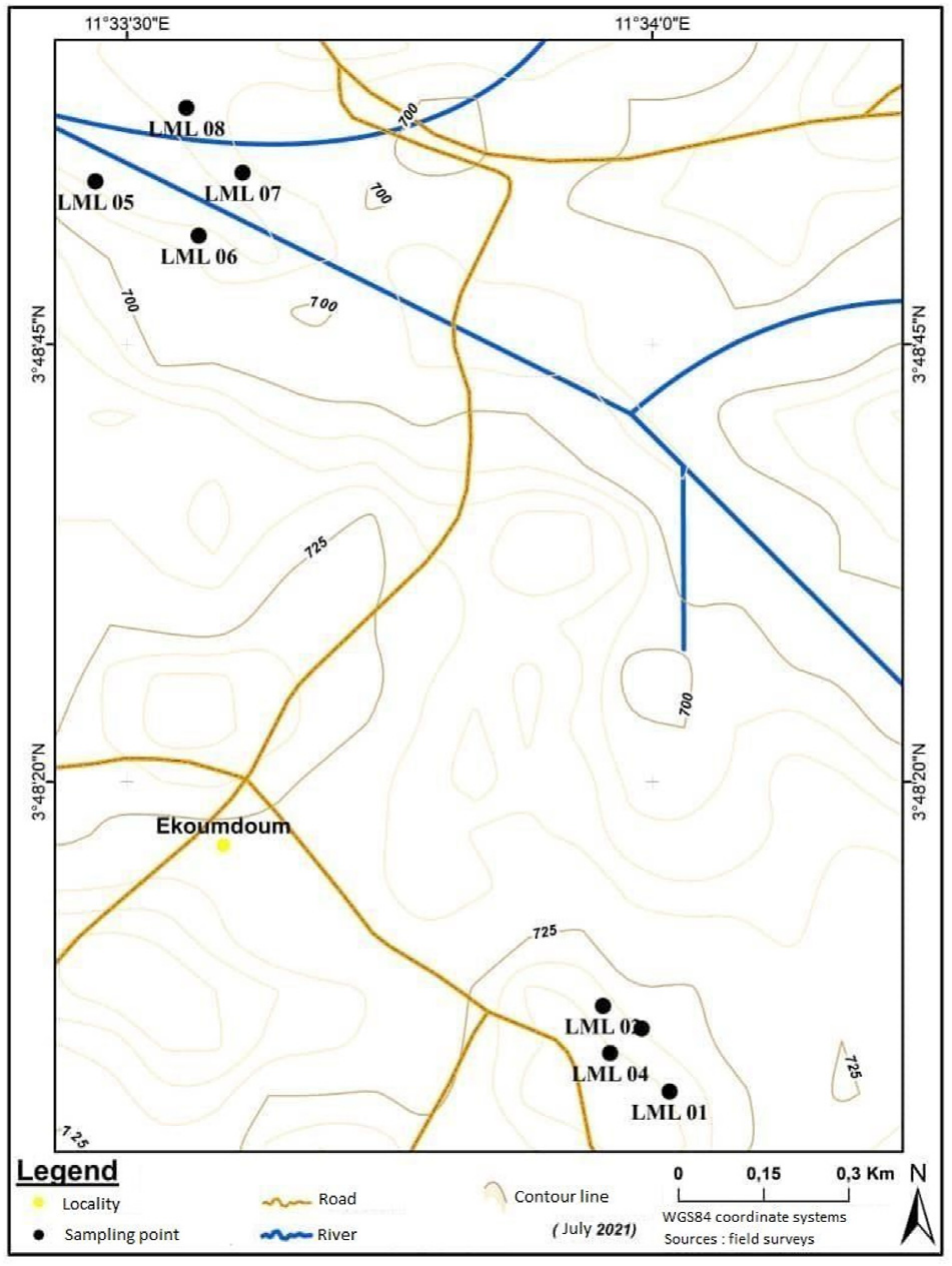
Sampling map.

The sites from which the samples were taken are sites used by the local population for backfilling buildings and secondary access roads. These sites are approximately 1.71 km apart as the crow flies. Approximately 40–50 kg of representative soil samples were collected from previously excavated soil layers. A sampling interval of approximately one hundred and fifty meters (150 m) between sampling points was used during sampling according to the grids defined on the sampling map (Figure 2). A total of eight (08) samples were collected, of which four (04) were taken at each study site. The geographic coordinates of the samples collected during this field phase are recorded in Table 1. Two soil profiles from the earthworks were observed and described at each sample site. These profiles show that the study area is mostly covered by clayey lateritic soils whose facies vary from one place to another. The sites are approximately 1.71 km apart as the crow flies.

**Table 1:** Geographical coordinates.

Sites	Samples	Latitudes (x)	Longitudes (y)	Altitudes (z)
**N°1**	LML 01	03°48′02.3″	011°33′01.0″	726
	LML 02	03°48′07.2″	011°33′57.2″	741
	LML 03	03°48′05.9″	011°33′59.4″	731
	LML 04	03°48′04.5″	011°33′57.6″	742
**N°2**	LML 05	03°48′54.3″	011°33′28.2″	684
	LML 06	03°48′51.2″	011°33′34.1″	685
	LML 07	03°48′54.8″	011°33′36.6″	680
	LML 08	03°48′58.5″	011°33′33.4″	682

### 3.1 Description of profile n°1 (Figure 3)

The profile of site n°1 presents four facies arranged on the horizon as follows.

- From 0 to about 0.3 m: a thin layer of dark-colored vegetal soil in which organic matter can be observed. This layer of soil constitutes the A horizon of the weathering profile of the study area.
- From 0.3 to 2 m deep: A layer of soil with a sandy-clay texture (determined by touch) of a reddish to whitish hue within which nodules of unconsolidated lateritic cuirass are observed.
- From 2 to 4 m: lateritic soil of red color with the presence of small nodules and boulders from the alteration of the parent rock. The whole constituted by the second and third layers constitutes the B horizon.
- After 4 m: the lateritic soil still shows in places the texture of the parent rock. Despite the visible weathering, some deformation structures of the parent rock can be observed. This layer constitutes the isalterite package and is the C horizon of the weathering profile observed at the study site.

### 3.2 Description of profile n°2 (Figure 3)

This profile presents four horizons whose layout is as follows:

- From 0 to 0.45 m deep: blackish-colored soil layer, covered with plants and constituted of organic matter. It is the organo-mineral layer that constitutes the A horizon.
- From 0.45 to 3 m depth: a layer of soil with nodules of more or less rounded shape and arranged in a decreasing manner according to the granulometry from top to bottom. – From 3 to 6 m depth: lateritic soil composed of more or less angular grains. The granulometric distribution decreases from top to bottom. It is essential to specify that on this layer, the structures constituting the mother rock are partially present and still observable. It extends over a depth of about three (3) meters and represents the isalterite layer.
- From 6 m depth: altered rock, the alteration made it less resistant.

## 4 Methods

### 4.1 X-ray fluorescence spectrometry (XRF)

Chemical analyses were obtained by XRF analysis, a Niton XL3t980 hXRF analyzer (equipped with a 50 kV X-ray tube with Ag anode and a silicon drift detector with an 8 mm spot) was used. The raw data were plotted as spectra, where the x-axes represent the element-specific fluorescence energy (unit keV) and the y-axes quantify the number of photons (unit cps) received by the detector. Detection is possible for most elements with atomic numbers ranging from 12 (magnesium) to 92 (uranium). 21 silicon-based standards, called certified reference materials (CRM), filled in cups and covered with a 4 μm polypropylene film were measured by a specific mode of the hXRF device (mining/mineral mode). The measured values were plotted using a trend line equation, and the “fit coefficient” R2 (correlation coefficients) was determined. Then, a classification was made based on the quality of the regression line and the distribution of the data. The raw soil powders were analyzed by XRF to obtain the proportion of major elements.

### 4.2 X-ray diffractometry (XRD)

Mineralogical analyses were obtained by XRD measurements that were performed using a Bruker- AXS D8 diffractometer equipped with a Lynx eye position-sensitive detector, with Cu.

Kα radiation ʎCu = 1.54056 Ǻ operated at 40 kV and 40 mA, an increment of 0.013° 2θ, and a measurement time in 30 s steps. Diffraction patterns were collected in the 2-theta range from 7.5° to 90°. Qualitative phase composition analysis of the powder samples was performed using.

PDF-2 2007 release software and X’Pert HighScore Plus. XRD was performed on the raw soils.

### 4.3 Physical analysis

The physical analyses were carried out in the geotechnical laboratory of MIPROMALO (Cameroon). The natural water content was determined according to the NF P 94-050 (1995) standard. The specific gravity was determined according to the NF P 94-054 (1991) standard. Dry particle size analysis was performed by sieving and sedimentation according to NF P 94-056 (1996) and NF P 94-057 (1992) standards respectively. The methylene blue test was carried out according to the NF P 94-068 (1998) standard. The compaction parameters (maximum dry density and optimum water content) were determined by the modified Proctor test according to the NF P 94-093 (1999) standard. The CBR index was evaluated according to the NF P 94-078 (1997) standard. The Atterberg limits were determined according to the NF P 94-051 (1993) standard. The absorption rate was determined by immersing the specimen in water for 24 h according to the ASTM standard.

### 4.4 Making and testing of compressed earth brick specimens

For the realization of the mechanical tests such as compression, traction by flexion, apparent density, and water absorption, it was necessary to make specimens for each study site. The procedure for manufacturing the test specimens is the one defined by the Cameroonian standard NC_102-115 (2007) which is as follows: After drying the samples in an oven at 105 °C, the lateritic soils were crushed and sieved to 2 mm. Stabilization was done with compound Portland cement (Dangote 3 × 42.5R) according to the standard at several percentages (0%, 4%, 6%, 8%, and 10%). The water content used corresponds to the Optimal Water Content (OWC) of the material. The total amount of cement-stabilized material per specimen is 100 g for dimensions of 8 × 4 × 1.5 cm and 140 g for dimensions of 4 × 4 × 4 cm. The compression was done with a mechanical press and molds corresponding to the dimensions mentioned above. Immediately after removal from the mold, the specimens were cured in plastic films to allow the chemical reactions of the cement to take place. The curing times observed are 7 days, 14 days, 28 days, and 56 days.

The bulk density and linear shrinkage of the manufactured brick specimens were determined according to NC_102-115 (2007). The standards NF P 94-420 (2000) and NF P 94-422 (2001) were used to measure the compressive and flexural strengths respectively.

### 4.5 Statistical analyses of the studied soil parameters and bricks

The statistical analyses aim to study the interaction between different parameters. In this work, these analyses were made using the XLStat software. The mean, standard deviation, variance, and median followed by the Pearson correlation test were carried out on the physicochemical and mechanical data. Those presenting strong correlations allowed to build prediction models of the mechanical behavior of compressed earth bricks.

## 5 Results and discussions

### 5.1 Chemical analysis

#### 5.1.1 X-ray fluorescence spectrometer (XRF) analysis

The results of the chemical analysis of major elements on the total fraction of lateritic materials are reported in Table 2. These analyses show that the soils in the study area are predominantly ferric laterites. The hematite content (Fe_2_O_3_ from 20.01 to 45.31%) is relatively high and predominates over the other elements present, silica (SiO_2_ from 20.82 to 38.73%) is present in moderate proportions, the alumina content (Al_2_O_3_ from 17.48 to 24.15%) is low to moderate. The loss on ignition is moderate and varies around 11%. The other oxides (CaO, MgO, SO_3_, K_2_O, Na_2_O, P_2_O_5_) are weakly represented with contents lower than 1.00%. According to their chemical composition, the studied lateritic materials are mainly composed of aluminosilicate and iron sesquioxide. The high presence of iron oxide in these soils explains the reddish color of the studied material. According to Autret (1980) classification of lateritic soils, the S/R ratio (0.32; 0.96) < 1.33 given by Eq. (1) is representative of true laterite. These S/R ratio values are close to that obtained by Nzabakurikiza et al. (2012) (0.57–1.24) on siliceous and ferric gravels from the Yokadouma locality in eastern Cameroon.

SR=SiO2RSO3withRSO3=Fe2O3+Al2O3

**Table 2:** Physico-chemical parameters.

Parameters	SITE N°1				SITE N°2			
	LML 01	LML 02	LML 03	LML 04	LML 05	LML 06	LML 07	LML 08
**TE (%)**	8,13	15,11	8,30	15,39	9,50	9,79	14,34	11,46
**G%**	48,83	34,84	50,97	19,89	38,41	30,84	27,34	40,09
**S%**	18,75	30,70	25,72	30,83	34,14	35,41	29,28	23,47
**L%**	5,40	6,70	1,08	8,62	2,75	3,37	4,23	5,33
**A%**	27,01	27,77	22,23	40,65	24,71	30,37	39,14	31,11
**Dr.**	2,50	2,55	2,61	2,35	2,73	2,37	2,41	2,42
**VBS**	1,00	1,07	0,80	1,33	0,60	0,93	0,87	0,93
**Ll**	63,37	62,88	76,08	61,89	58,03	52,89	49,90	54,95
**Lp**	43,02	38,94	35,84	36,49	31,16	27,30	24,85	30,00
**Ip**	20,36	23,94	40,24	25,40	26,87	25,59	25,04	24,95
**MO%**	4,78	3,54	5,09	4,58	6,43	5,31	5,27	5,04
**Wopm (%)**	13,00	12,50	11,50	12,90	12,90	11,80	15,80	12,70
**γsmax(t/m3)**	1,87	2,07	2,04	1,95	2,08	2,11	1,98	2,06
**ICBR**	15,00	20,00	30,00	20,00	32,00	35,00	20,00	20,00
**SiO**_**2**_**(%)**	20,82	35,03	32,14	34,21	30,97	35,94	38,73	32,42
**Al**_**2**_**O**_**3**_**(%)**	19,68	24,15	17,48	18,06	22,02	20,42	20,16	21,32
**Fe**_**2**_**O**_**3**_**(%)**	45,31	24,28	32,53	30,46	28,73	26,37	20,01	28,93
**CaO (%)**	0,79	0,07	0,37	0,14	0,03	0,01	0,04	0,05
**MgO (%)**	0,02	0,07	0,11	0,08	0,06	0,01	0,07	0,01
**SO**_**3**_**(%)**	0,04	0,06	0,06	0,05	0,06	0,03	0,04	0,04
**K**_**2**_**O (%)**	0,03	0,56	0,57	0,39	0,44	0,12	0,62	0,13
**Na**_**2**_**O (%)**	0,09	0,00	0,02	0,08	0,15	0,09	0,13	0,01
**P**_**2**_**O**_**5**_**(%)**	0,47	0,12	0,18	0,27	0,28	0,16	0,10	0,13
**PaF (%)**	12,75	12,90	11,60	13,18	12,54	12,05	12,46	13,30
**S/R**	0,32	0,72	0,64	0,71	0,61	0,77	0,96	0,65
**CIA**	95,58	97,46	94,79	96,73	97,26	98,93	96,23	99,12

The SiO_2_/Al_2_O_3_ ratio <2 suggests that the rock from which these soils were derived was not very high in silica content. The CIA (Chemical Index of Alteration) = (100 × Al_2_O_3_/Al_2_O_3_ + CaO + NaO + K_2_O) values are all in the range of 94.79–99.12%. These values according to Nesbitt and Young (1982) are due to the presence of kaolinite and even gibbsite in these materials. The prevailing pedogenetic process is monosiallitization which favors the formation of kaolinite (Pédro, 1966).

Chemical analyses report that iron and aluminum sesquioxides (Al_2_O_3_+Fe_2_O_3_) represent between 44.17% and 50% of the weight of the studied laterites, i.e. about half of the major elements represented in the studied soils.

#### 5.1.2 Organic matter content

From the point of view of construction or earth brick production, organic matter particularly reduces the mechanical strength of building materials. They cause corrosion, softening of the material in time, and harm the stabilization process of cement Guillaud and Hermann (1989). Their proportions in the studied soils vary between 3.54% and 6.44%. Organic matter is one of the most important factors affecting the plasticity properties of soils (De Oliveira Modesto and Bernardin, 2008; Hajjaji et al., 2010) since organic matter increases water absorption causing a high plasticity index. Depending on the low proportion observed in the studied soils, the negative effect of organic matter may be negligible.

### 5.2 Mineralogical analysis

X-ray diffraction analyses performed on the samples from the study area shown in Figure 4 that the mineral composition is mainly quartz, illite, hematite, kaolinite, goethite, gibbsite, muscovite, magnesite, and trace minerals are gypsum, halloysite, Boehmite, rutile, pyroxene, montmorillonite. Illite improves the plasticity of the material (Nzeukou Nzeugang et al., 2021), montmorillonite is clay with absorption and swelling properties; this mineral absorbs much more than other clay minerals like kaolinite or illite (Wesley, 1973; Guorui, 1996; Ngon Ngon et al., 2012). This phenomenon of absorption can lead to an increase in plasticity (Abdullah Sabtan, 2005) and a significant shrinkage during drying. The mineralogical composition shows that montmorillonite is not present in large proportions, so the harmful influence that could be caused by the presence of this mineral is negligible. On the other hand, the kaolinite present in greater proportion in the material gives it certain workability increasing the molding properties (Morin and Todor, 1975) which are important for application in compressed earth bricks. The presence of hematite in this material is related to the presence of iron oxide (29.57 on average). Boehmite and gibbsite are both due to the relatively high aluminum oxide content (average 20.41) observed in these soil samples. The presence of goethite and other iron oxides existing in these residual soils may act as cementitious agents, which may contribute to tensile cracking by making the compacted structure relatively brittle (Kamtchueng et al., 2015).

**Figure 3:**
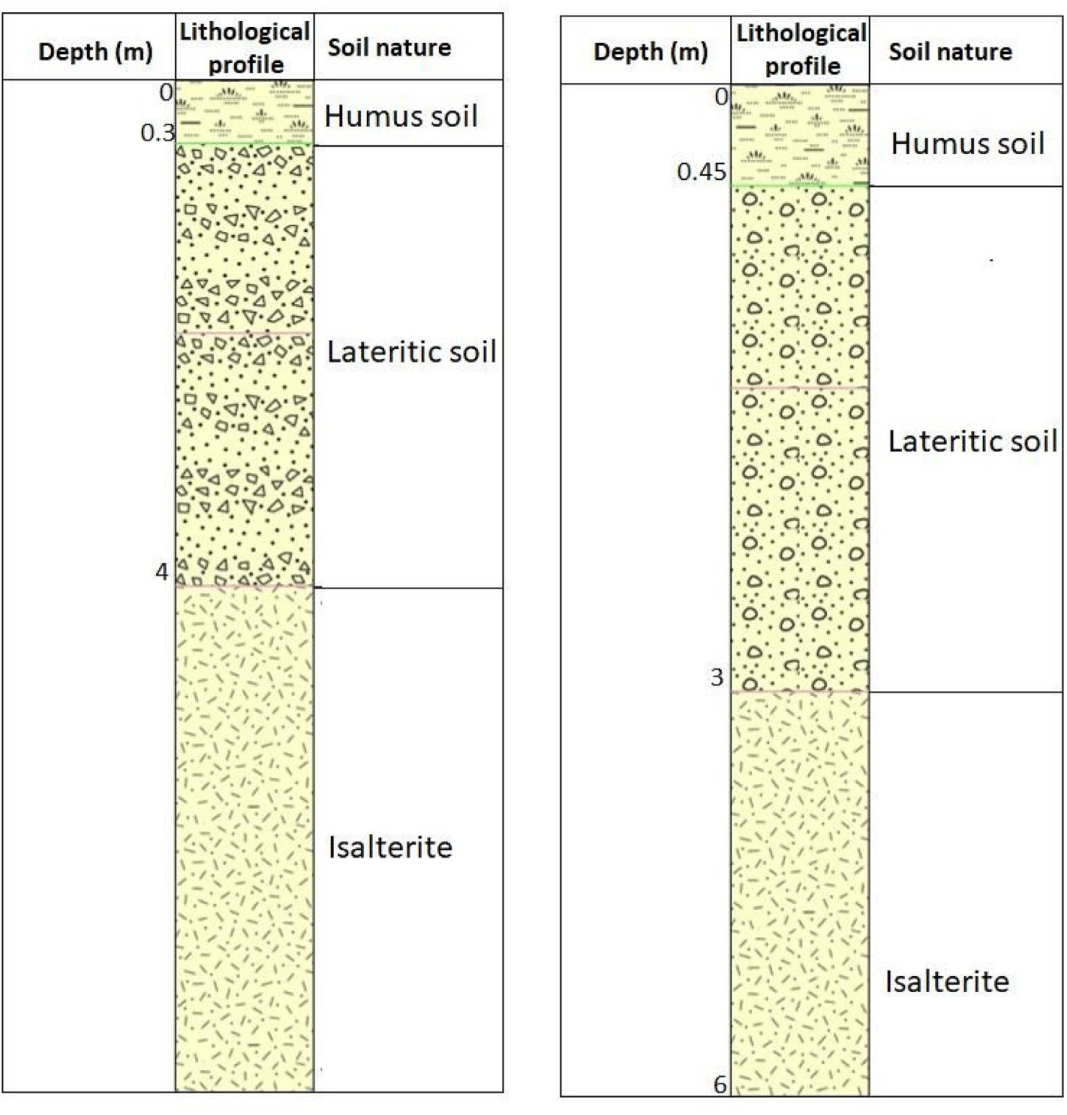
Lithological profile.

**Figure 4:**
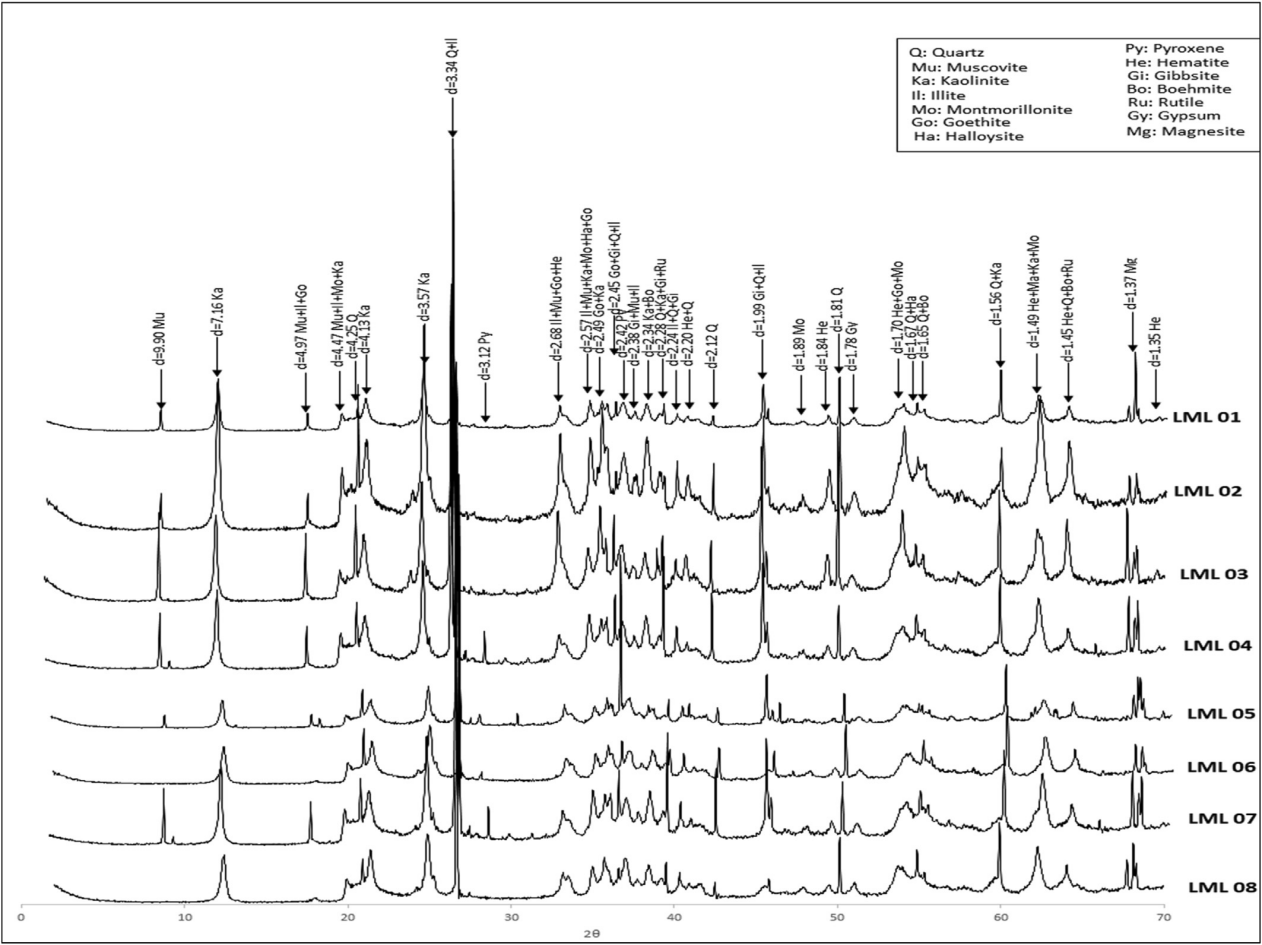
Mineralogy.

### 5.3 Physical and mechanical properties of the studied soils

#### 5.3.1 Natural water content

The analysis of the natural water content obtained is recorded in Table 2. These values range from 8.1% to 15.4%, with an average of 11.73%. Some of the relatively high values of water content can be explained by the fact that the sampling campaigns were carried out at different sites in April, at the beginning of the rainy season. Different factors have a significant effect on the natural water content such as the depth of the well, organic matter, and the nature of the soils which have a water retention capacity depending on their mineral or clay particle content (Ben Abdelghani et al., 2014). These results show that soils with high water content may reflect a high proportion of clay and silt particles in the corresponding soil. Furthermore, the natural water content values of the studied soils show that compared to their respective optimum water content, these soils were mostly in a dry state at the sampling period except for the LML 02 and LML 04 samples which were in a wet or muddy state.

#### 5.3.2 Grain size distribution

**Figure 5:**
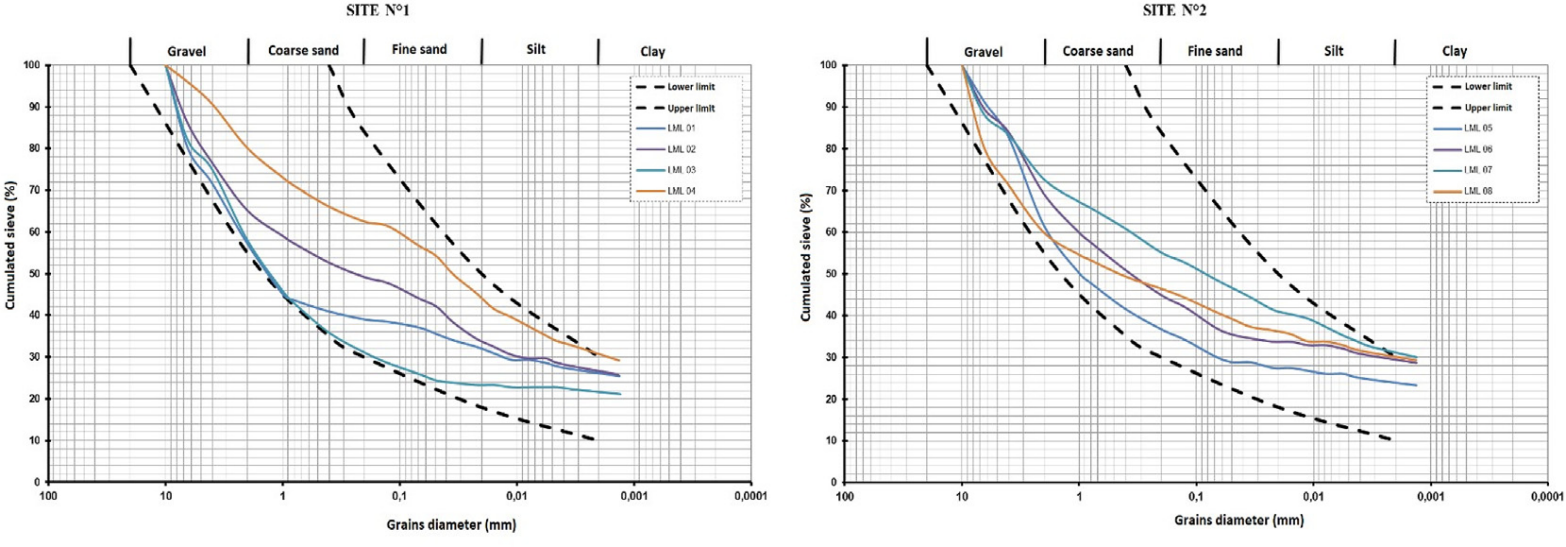
Grains size distribution.

The results of the particle size distribution analysis are reported in Table 2. The values are projected into the particle size envelope of the soil materials used for the production of compressed earth bricks (CEB) according to NC_102-115 (2007) and CRAterre Figure 5. The results show that the soil samples have a high content of gravel that reaches 50.97% for sample LML 03, while the content of sand and silt is respectively between 18.75%–35.40% and 1.08%–8.62%, the clay content is between 22.23% and 40.65%. According to these results, the studied soils are mainly composed of gravel followed by sand, except for LML 04 and LML 07 which contain more clay particles. These soils can then be classified as Group B fine-grained soils according to the French road grading manual (GTR). The Pearson correlation matrix shown in the table indicates that there is a strong correlation between the natural water content (W) with the clay content (A%) and the silt content (L%). Increasing clay and silt content tends to increase the natural water content of these soils. The correlation coefficient of Pearson values is 0.75 between natural water content and clay, and 0.73 between natural water content and silt. The authors (Holtz and Kovacs, 1981) have shown that particle size distribution can have a significant effect on soil plasticity. Since the presence of certain swelling clay minerals can increase water absorption. The particle size distribution of raw materials can also influence technological parameters such as mechanical strength and durability (Guettala et al., 2002; Carmen Jime Nez Delgado and Ignacio, 2005) since the compressive strength of mud bricks increases with a high proportion of sand content, the best results are obtained for a sand proportion between 40 and 65%.

#### 5.3.3 Specific weight

The average specific gravity values are 2.51 and 2.48, respectively, for the soils at Site #1 and Site #2. The higher specific gravity values indicate that the soils studied are very heavy with coarse textured fragments (Nzeukou Nzeugang et al., 2021). Chemical composition has an influence on the specific gravity of laterites, lower specific gravity values (2.48) would characterize high alumina content (20.98 wt%) while higher specific gravity values (2.51) would be due to high iron content (33.14 wt%) (Maignien, 1958). These results are in agreement with the research of (Mahalinga-Iyer and Williams, 1985) who showed that specific gravity values of lateritic soils in the equatorial zone are generally between 2.5 and 3.6.

#### 5.3.4 Methylene blue values

The values of methylene blue of the studied soils are globally included between 0,6 and 1,34 which indicates that all the studied soils are weakly sensitive to water. The interpretation of methylene blue values according to the standard (NF P94 068), shows that the soils studied are essentially silty. The relatively low values may be due to the relatively low content of clay particles in the particle size distribution. The correlation matrix of the data presented in Table 3 shows a good correlation between the methylene blue value and the silt content with a correlation coefficient of 0.87 and a relatively good correlation with the clay content (0.61). These values show that silt content has a more significant effect on the methylene blue value. They also show that the most active fraction in these soils is the silt fraction. The methylene blue values obtained can also express the swelling potential of these soils, low values indicate that in the mineralogical composition of these soils, swelling clay is poorly represented.

**Table 3:** Pearson correlation matrix between physicochemical and mineralogical parameters.

W	1,00																							
**G%**	**-0,80**	**1,00**																						
**S%**	0,32	-0,62	**1,00**																					
**L%**	**0,73**	-0,59	-0,08	**1,00**																				
**A%**	**0,75**	**-0,86**	0,18	0,64	**1,00**																			
**Dr**	-0,44	0,59	0,04	-0,54	**-0,79**	**1,00**																		
**VBS**	0,60	-0,51	-0,12	**0,87**	0,61	-0,70	**1,00**																	
**Ll**	-0,33	0,58	-0,34	-0,21	-0,57	0,47	0,05	**1,00**																
**Lp**	-0,13	0,42	-0,52	0,35	-0,35	0,25	0,39	0,70	**1,00**															
**Ip**	-0,32	0,37	0,07	-0,65	-0,42	0,38	-0,35	0,66	-0,08	**1,00**														
**MO**	-0,53	0,11	0,25	-0,62	-0,17	0,35	**-0,74**	-0,27	-0,54	0,20	**1,00**													
**OMC**	0,46	-0,41	-0,04	0,20	0,63	-0,24	-0,03	-0,59	-0,39	-0,41	0,12	**1,00**												
**MDD**	0,00	-0,07	0,66	-0,39	-0,30	0,23	-0,42	-0,17	-0,52	0,31	0,19	-0,38	**1,00**											
**ICBR**	-0,42	0,05	0,68	-0,69	-0,40	0,33	-0,55	0,03	-0,46	0,53	0,58	-0,47	**0,73**	**1,00**										
**SiO**_**2**_	0,63	-0,67	0,69	0,00	0,48	-0,31	0,05	-0,39	**-0,72**	0,21	-0,04	0,26	0,58	0,32	**1,00**									
**Al**_**2**_**O**_**3**_	0,27	-0,06	0,27	0,17	-0,19	0,26	-0,19	-0,40	-0,06	-0,49	-0,19	0,08	0,47	-0,06	0,14	**1,00**								
**Fe**_**2**_**O**_**3**_	-0,62	0,61	-0,69	0,03	-0,39	0,16	0,11	0,50	**0,74**	-0,08	-0,01	-0,36	-0,63	-0,29	**-0,96**	-0,37	**1,00**							
**CaO**	-0,53	0,64	**-0,79**	-0,03	-0,34	0,13	0,11	0,54	**0,74**	-0,03	-0,16	-0,14	**-0,76**	-0,42	**-0,87**	-0,41	**0,91**	**1,00**						
**MgO**	0,26	-0,04	0,16	-0,13	-0,03	0,37	0,00	0,60	0,16	0,66	-0,12	0,05	-0,04	0,08	0,30	-0,32	-0,22	-0,04	**1,00**					
**SO**_**3**_	0,10	0,22	0,12	-0,09	-0,42	**0,76**	-0,18	0,65	0,41	0,47	-0,10	-0,23	0,15	0,08	-0,01	0,14	-0,04	-0,03	**0,76**	**1,00**				
**K**_**2**_**O**	0,46	-0,21	0,36	-0,15	0,08	0,34	-0,16	0,22	-0,16	0,48	-0,09	0,31	0,19	0,10	0,58	0,04	-0,60	-0,37	**0,88**	0,67	**1,00**			
**Na**_**2**_**O**	-0,12	-0,30	0,34	-0,17	0,26	0,09	-0,34	-0,48	-0,37	-0,27	0,71	0,52	-0,21	0,22	-0,02	-0,11	-0,04	-0,06	-0,08	-0,21	0,00	**1,00**		
**P**_**2**_**O**_**5**_	-0,47	0,34	-0,45	0,14	-0,21	0,20	0,09	0,28	0,65	-0,30	0,14	-0,13	-0,70	-0,26	**-0,90**	-0,28	**0,89**	**0,79**	-0,19	-0,01	-0,50	0,34	**1,00**	
**PaF**	0,55	-0,38	-0,20	**0,84**	0,48	-0,32	0,53	-0,36	0,19	**-0,71**	-0,33	0,26	-0,25	-0,68	-0,11	0,37	0,03	-0,11	-0,35	-0,08	-0,27	-0,13	0,12	**1,00**
**Variables**	**W**	**G%**	**S%**	**L%**	**A%**	**Dr**	**VBS**	**Ll**	**Lp**	**Ip**	**MO**	**OMC**	**MDD**	**ICBR**	**SiO**_**2**_	**Al**_**2**_**O**_**3**_	**Fe**_**2**_**O**_**3**_	**CaO**	**MgO**	**SO**_**3**_	**K**_**2**_**O**	**Na**_**2**_**O**	**P**_**2**_**O**_**5**_	**PaF**

#### 5.3.5 Atterberg limits

**Figure 6:**
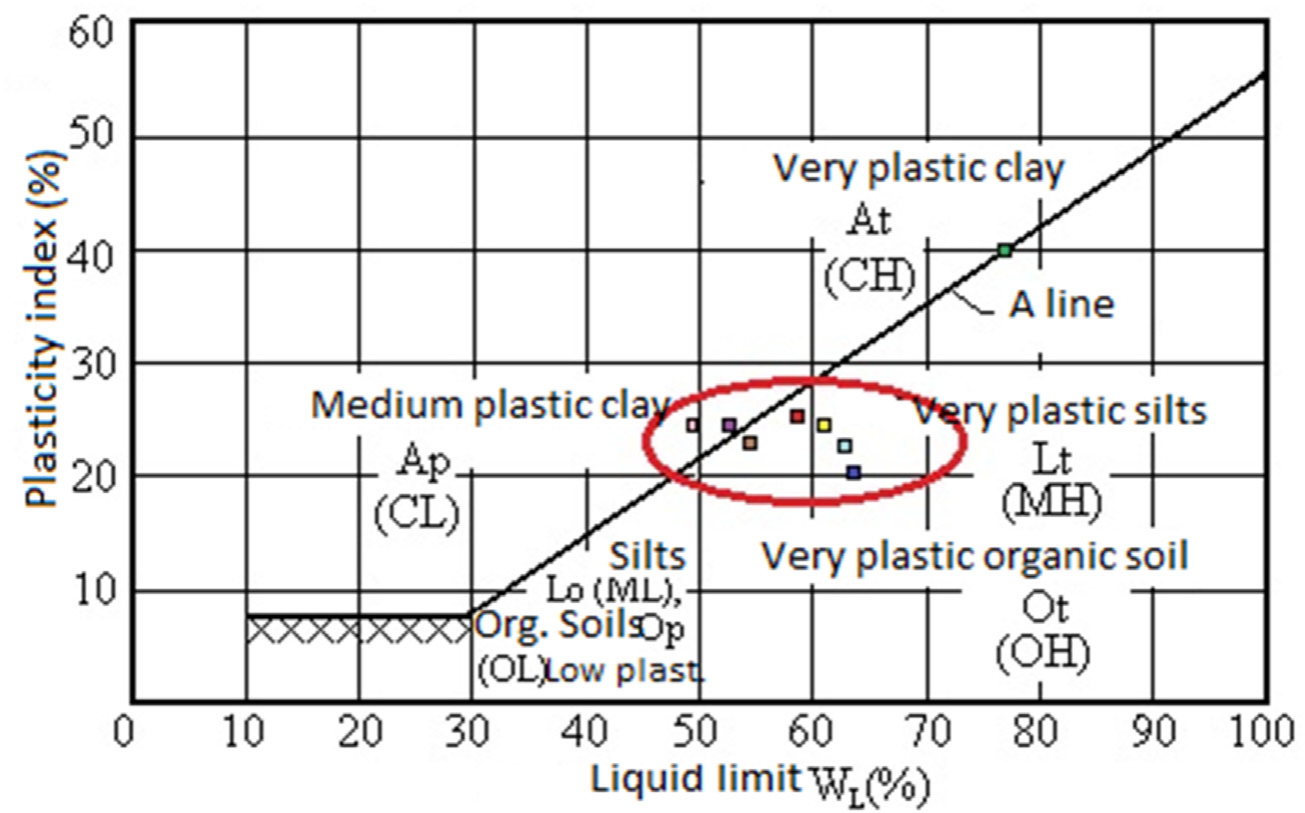
Atterbergs limit.

Observation of the plasticity chart data (Figure 6) shows that the majority of the lateritic soils in the study area are in the low plastic clay to high plastic silt zone, except sample LML 03 which is in the high plastic clay zone. Samples LML 06 and 07 tend to be closer to the low plastic clays. The main factors influencing soil plasticity are the quantity and quality of the clay fraction (CRATERRE). Concerning the quality of the clay, a good correlation is established between the liquidity limit with CaO and Fe_2_O_3_. Indicating that these elements are the main elements affecting the plasticity of these soils, these elements would thus be the main constitutive elements of the clays in the studied soils. The table shows 3 that the chemical composition of these soils has a significant effect on the consistency of these materials. The proportions of silica SiO_2_ show a good correlation with the liquid limit (−0.72), indicating that the increase in SiO_2_ content tends to cause a decrease in the liquid limit explained by the low water retention capacity of quartz. The NC_102-115 (2007) standard on the manufacture of compressed earth bricks recommends that to obtain good mechanical properties, soils should have values between 25 and 50 for the liquid limit, 20 to 35 for the plastic limit, and 2 to 30 for the plasticity index. Overall, the samples in the study area fall within these ranges. For soils in the study area that do not fall within these ranges, especially those with very high plasticity, the addition of cement will improve their mechanical properties for the manufacture of CEBs. Plasticity specifications according to CEBTP (1984) indicate that the plasticity index (PI) values should be between 20 and 30% for use in subbase and less than 15% for use in base course. The values of the plasticity index in the study area oscillate around the average (26.55%), which suggests the possibility of their use as a sub-base.

#### 5.3.6 Compaction tests

Proctor tests performed on representative samples from each sampling site show that for the first site, the maximum dry densities (γdmax) range from 1.87 to 2.07 t/m^3^ for an average of.

**Figure 7:**
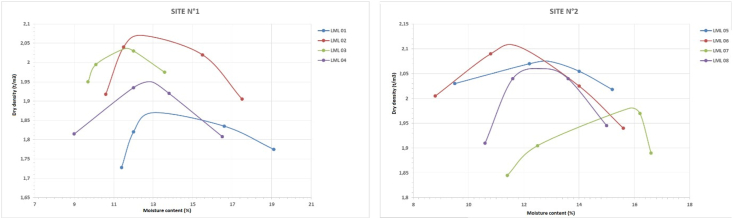
Proctor curves.

1.98 t/m^3^ and while the corresponding optimum water contents (Wopm) range from 11.5 to 12.9% for an average of 47.12%; For the second site, the maximum dry density (γdmax) varies from 1.98 to 2.105 t/m^3^ for an average of 2.05 t/m^3^ and while the optimal water content (Wopm) varies from 11.8 to 15.8% for an average of 13.3%. For better compaction of these soils, the moisture content values used should correspond to the results obtained by the modified Proctor test. The use of optimal water contents allows lubrication and rearrangement of the grains and minimizes the presence of voids in the material, which has the effect of densifying the BEC and thus increasing the mechanical strengths of the compacted soils. This water content must not be too high either, as the voids would be filled with water and therefore incompressible. On the other hand, if the water content is very low compared to the optimum water content, the soil is difficult to compact. The Proctor curves drawn from the data obtained are presented in Figure 7. According to CRATERRE, the bell-shaped curves show the typical textures of compressed soil blocks and the very flattened curves represent soils containing plastic clays so the compaction of the material is very much influenced by the water content. The Proctor curves of the studied soils show average curvatures; this translates that these soils can be used in the manufacture of stabilized BTC as well as in the realization of road works in embankment layers.

#### 5.3.7 Californian bearing ratio (CBR)

The CBR test is the most widely used test for determining the load capacity of soils. It is also used for the design of pavement layers. The bearing capacity indices at 95% of the Proctor optimum range from 15 to 30 for the first site with an average of 21.25 and from 20 to 35 for the second with an average of 26.75. According to the Recommendations for the use of lateritic gravels in pavements in Cameroon (DEGN, 1987), for CBR values at 95% of the Proctor optimum between 15 and 30, the soils studied are considered as class S4 soils useable as upper rail layer and as sub-base layer for T1 traffic. The CBR values can also provide information on the use of soils in building construction. Indeed, it gives information on the granulometric composition of the soil. Generally, the lower the CBR value, the higher the fine fraction of the studied soil. This observation can be supported by the Pearson correlation matrix (Table 3) which shows a more or less good correlation (−0.69) between the CBR index (ICBR) and the silt content. The CBR index of the studied soil tends to decrease when the percentage of silt increases. Moreover, this table also shows that there is a good correlation between the maximum dry density and the bearing capacity index (ICBR), the greater the maximum dry density the greater the bearing capacity of the soil with a correlation coefficient of 0.73 between the two parameters.

### 5.4 Properties of the compressed earth brick specimens

#### 5.4.1 Mechanical properties

##### 5.4.1.1 Compressive strength

**Figure 8:**
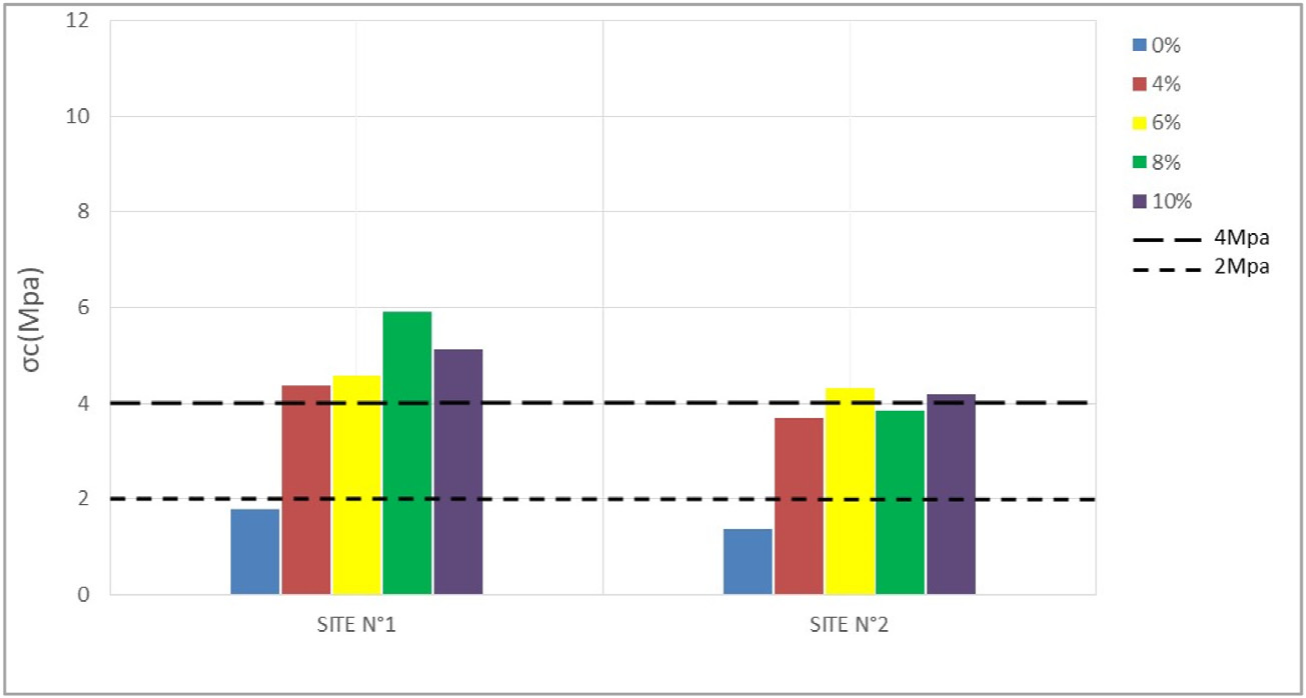
7-days **c**ompressive strength

**Figure 9:**
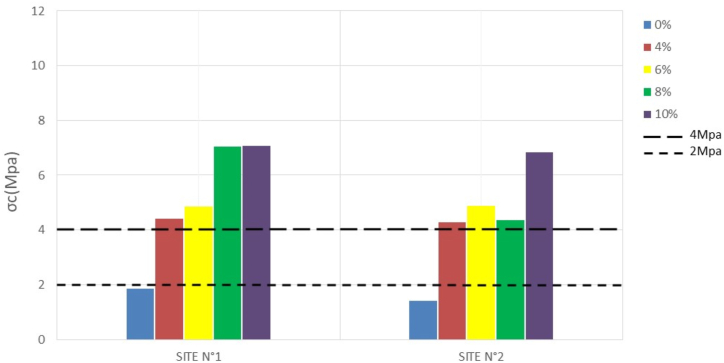
14-days compressive strength

**Figure 10:**
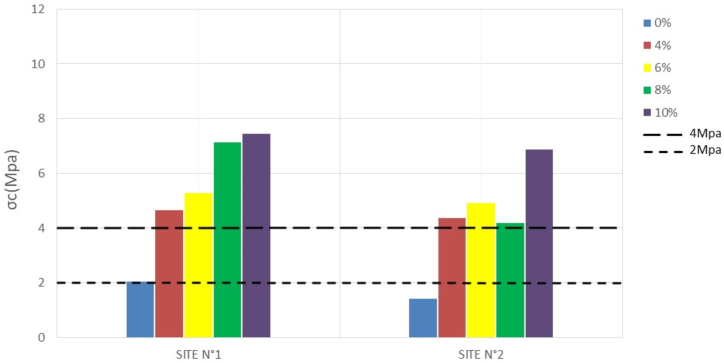
28-days compressive strength

**Figure 11:**
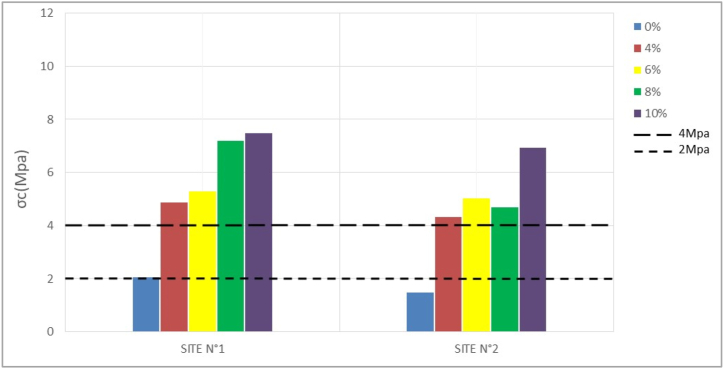
56-days compressive strength

The compressive strength (σc) of compressed bricks is one of the most important mechanical parameters to consider for placement. It is generally a function of the stabilization rate and the nature of the material (Kwon et al., 2010). Table 4 and the histograms in Figures 8, 9, 10, and 11 show the results obtained after compression and their variation with stabilization rates, respectively.

**Table 4:** Compressive strength.

SITES		σ_c_ (Mpa)			
		7 days	14 days	28 days	56 days
**N°1**	0%	1,78	1,86	2,05	2,06
	4%	4,37	4,42	4,65	4,86
	6%	4,58	4,87	5,27	5,29
	8%	5,91	7,05	7,13	7,18
	10%	5,12	7,08	7,45	7,47
**N°2**	0%	1,38	1,40	1,42	1,48
	4%	3,70	4,29	4,36	4,33
	6%	4,32	4,87	4,91	5,02
	8%	3,84	4,35	4,19	4,69
	10%	4,18	6,83	6,87	6,94

At 0% stabilization, the strengths obtained for a duration of 7–58 days vary between 1.7 to 2.06 Mpa per sample for the first site and 1.3–1.5 Mpa per sample for the second site. Since the standard lower limit of compressive strength of unstabilized mud bricks according to NC_102-115 (2007) is 2Mpa, site n°1 presents some acceptable values, while site n°2 presents no acceptable compressive strength value according to the standard, and this until 58 days. This could be due to the proportion of clay particles which tends to be higher on the samples from site N°1 than on those from site N°2. Indeed, in the unstabilized state, the clay present in the material can act as a natural binder and increase the mechanical strength of the compressed earth bricks. A proportion of clay between 15 and 30% is necessary to obtain good mechanical properties (Kwon et al., 2010).

Stabilization at 4, 6, 8, and 10 percent then yields much higher strengths at both sites that change with the rate of stabilization. According to NC_102-115 (2007), the compressive strength values of stabilized BECs should be between 4 and 12 Mpa. Both sites give results that comply with this standard. The 28-day strengths range from 4.65 to 7.45 Mpa for the former, and 4.36–6.87 Mpa for the latter.

The evolution of the strengths shows that at 0% the compressive strength hardly changes over time. The constancy of the mechanical strengths over time highlights the fact that after a period of 14–28 days, the optimum strength of the compressed earth bricks can be reached. Whereas with the addition of cement, a significant increase in strength is observed between 7 and 14 days. This increase in strength tends to stabilize after 28 days.

##### 5.4.1.2 Resistance to bending

**Figure 12:**
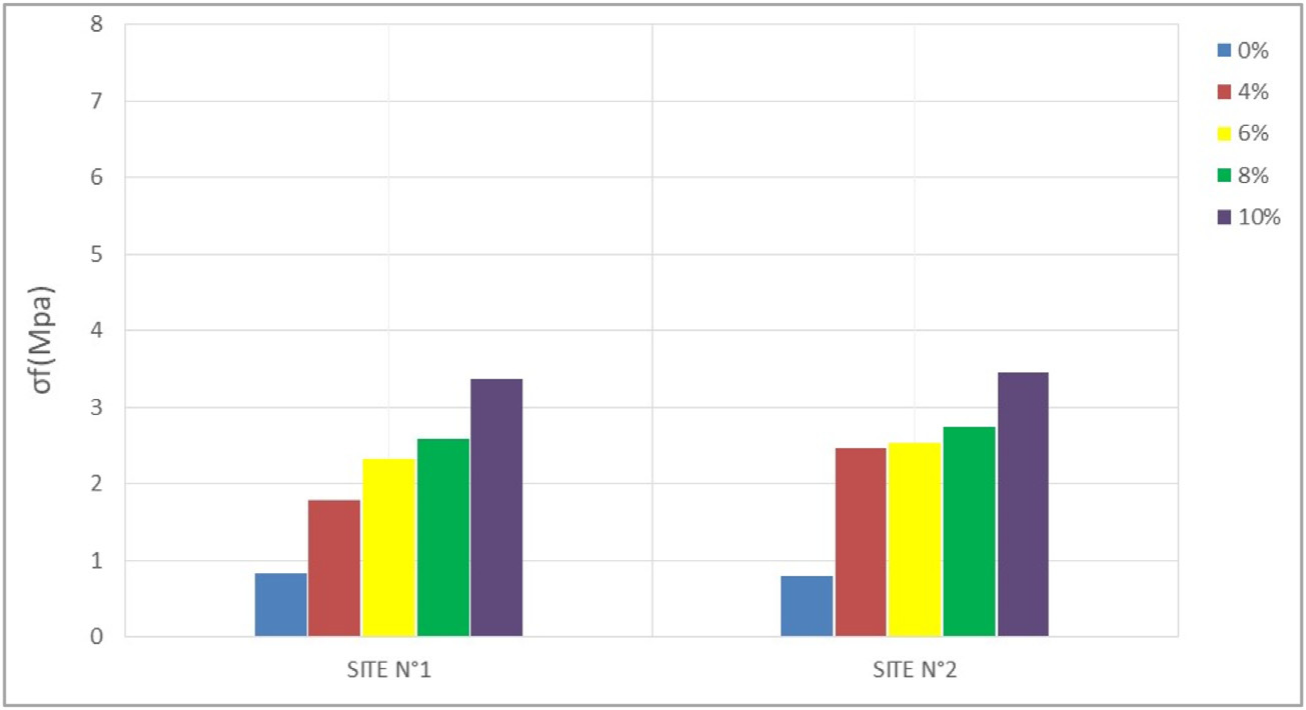
7- days bending tensile strength.

**Figure 13:**
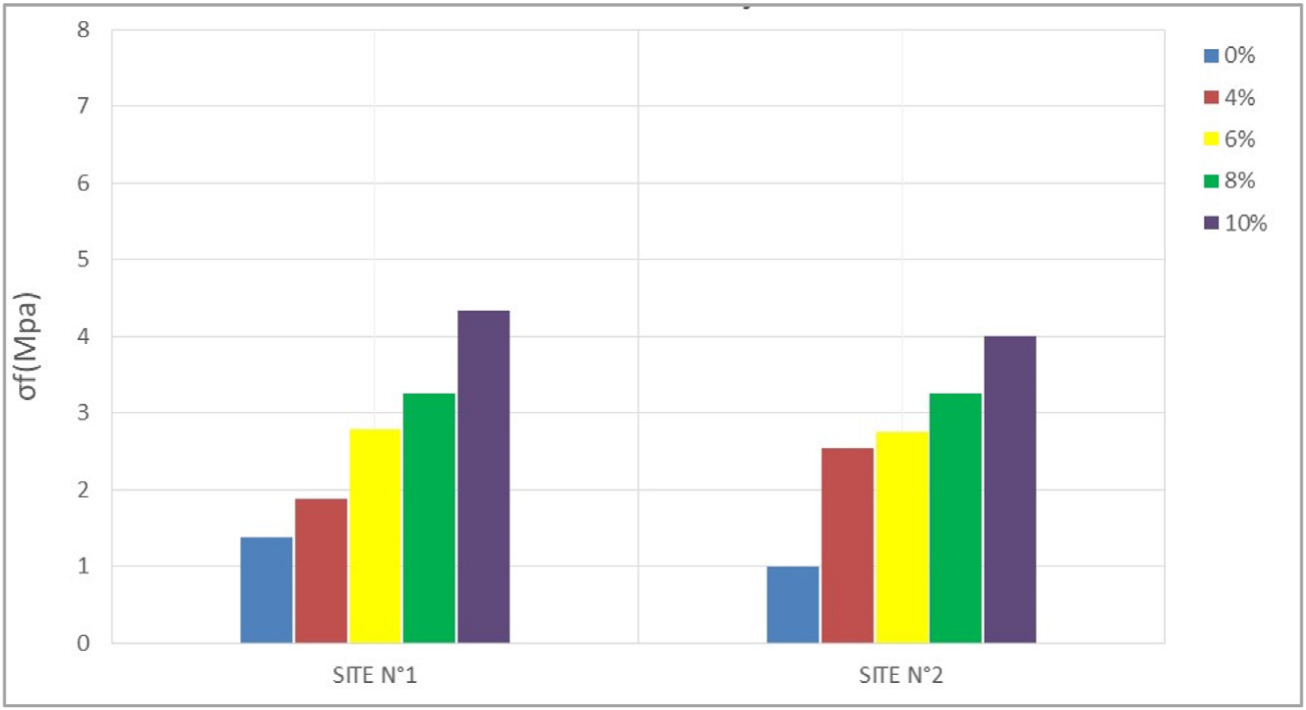
14-day bending tensile strength

**Figure 14:**
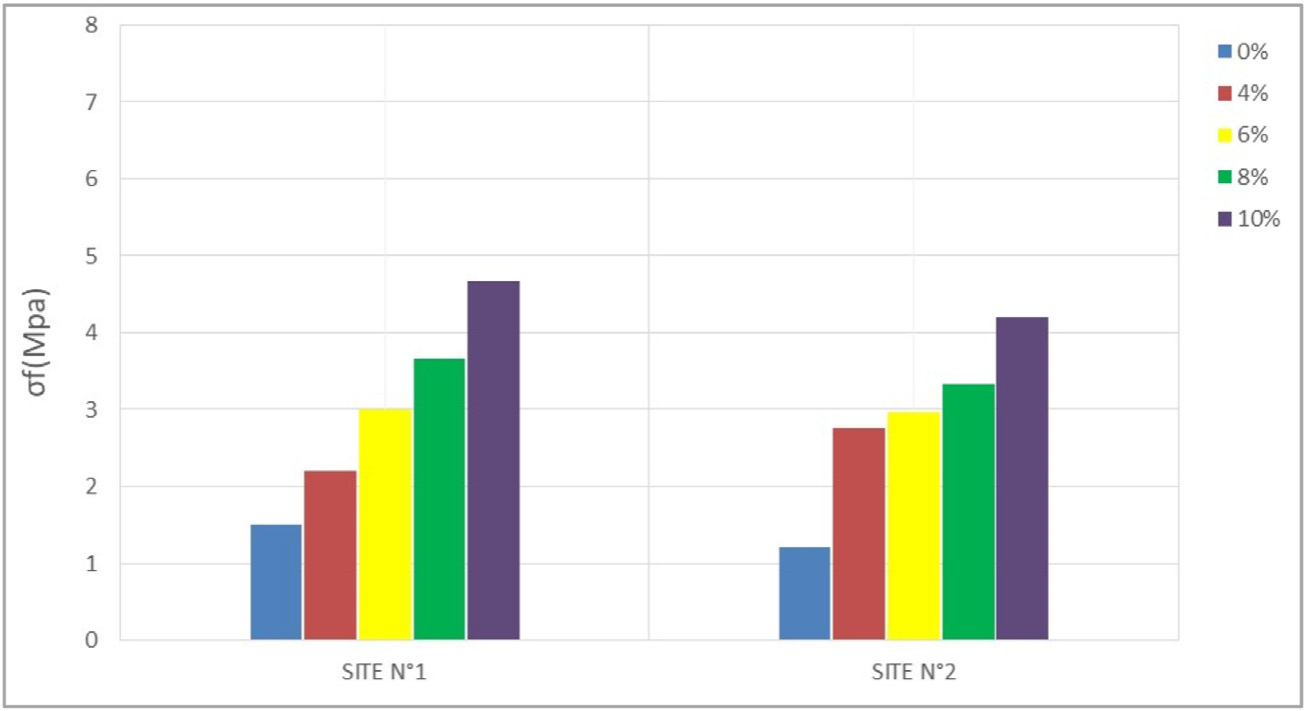
28- days bending tensile strength

**Figure 15:**
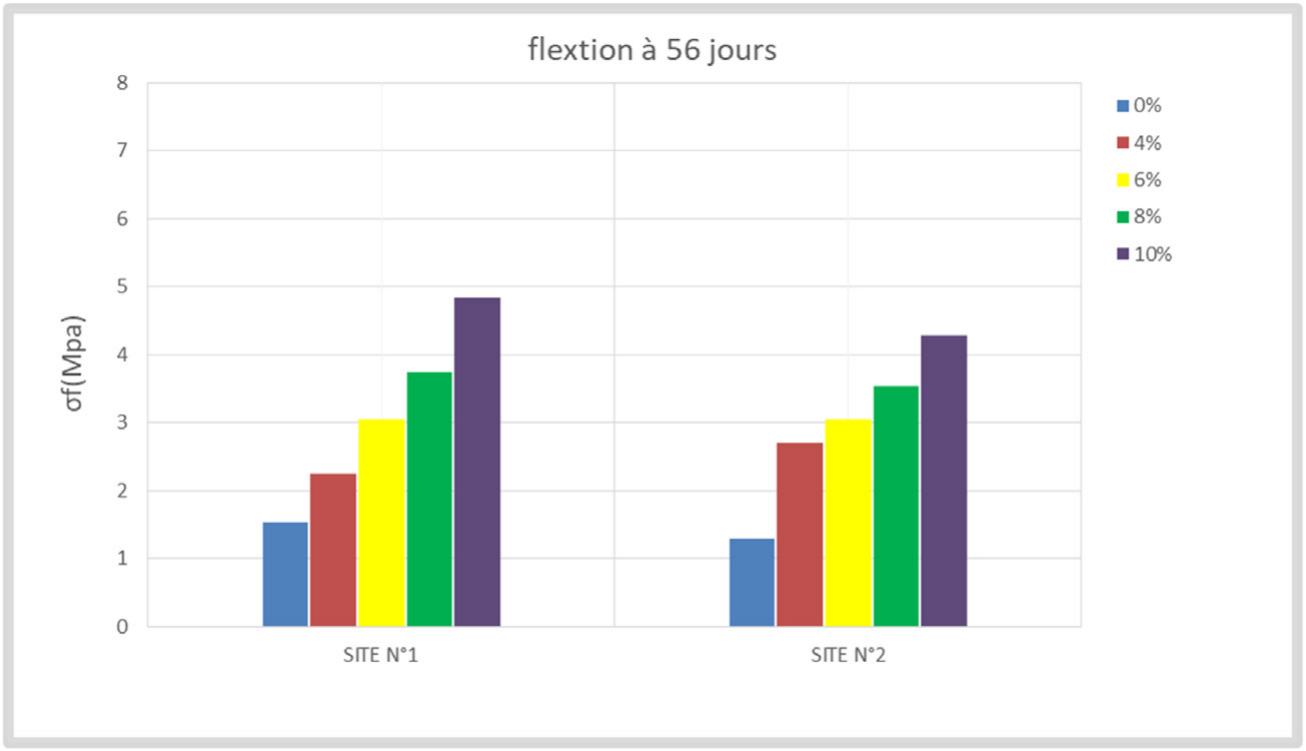
56- days bending tensile strength

The bending tensile test is a parameter that has been studied because of the potential bending stress of the compressed earth bricks in some structures. The results obtained are recorded in Table 5 and transcribed in the histograms of Figures 12, 13, 14, and 15. The resistance values evolve with time; the values observed at 28 days are higher than those observed at 14 days, which are themselves higher than those of 7 days. At 28 days, these values are between 1.5 and 4.66 Mpa for site n°1, 1.21, and 4.21 Mpa for site n°2 depending on the stabilization rates (4%, 6%, 8%, and 10%).

**Table 5:** Flexural strength.

SITES		σ_f_ (Mpa)			
		7 days	14 days	28 days	56 days
**N°1**	0%	0,83	1,38	1,50	1,54
	4%	1,79	1,88	2,21	2,25
	6%	2,33	2,79	3,00	3,04
	8%	2,58	3,25	3,66	3,75
	10%	3,38	4,33	4,66	4,83
**N°2**	0%	0,79	1,00	1,21	1,29
	4%	2,46	2,54	2,75	2,71
	6%	2,54	2,75	2,96	3,04
	8%	2,75	3,25	3,33	3,54
	10%	3,46	4,00	4,21	4,29

#### 5.4.2 Water absorption

**Figure 16:**
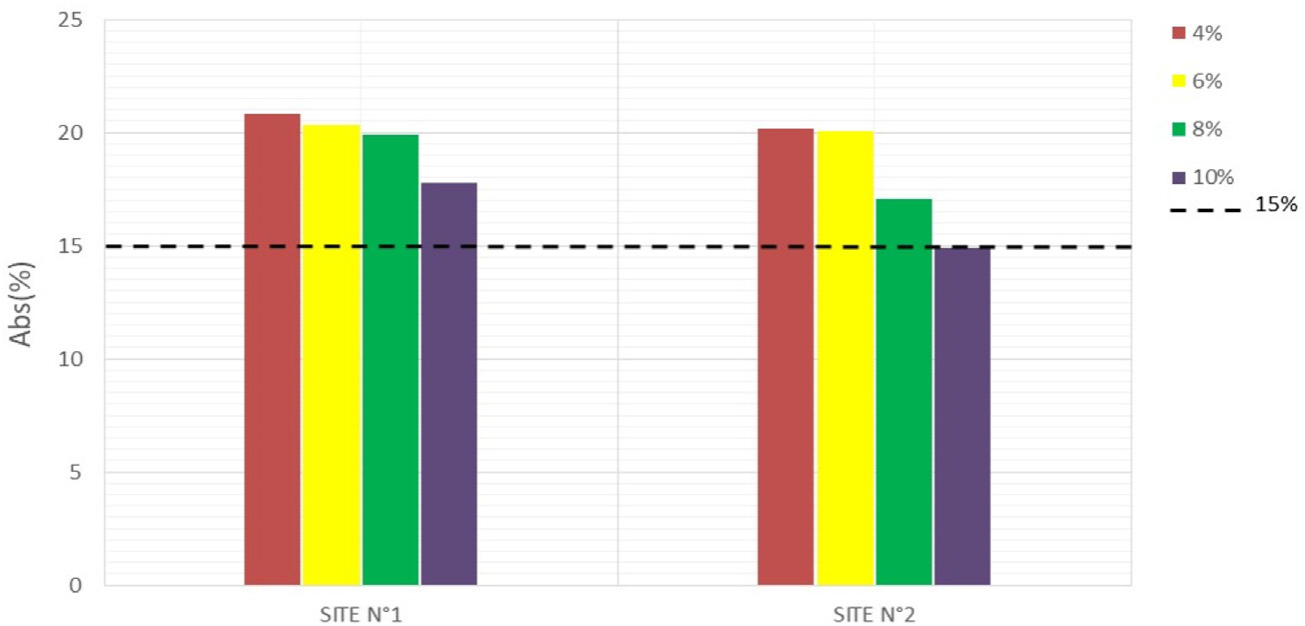
7-days water absorption

**Figure 17:**
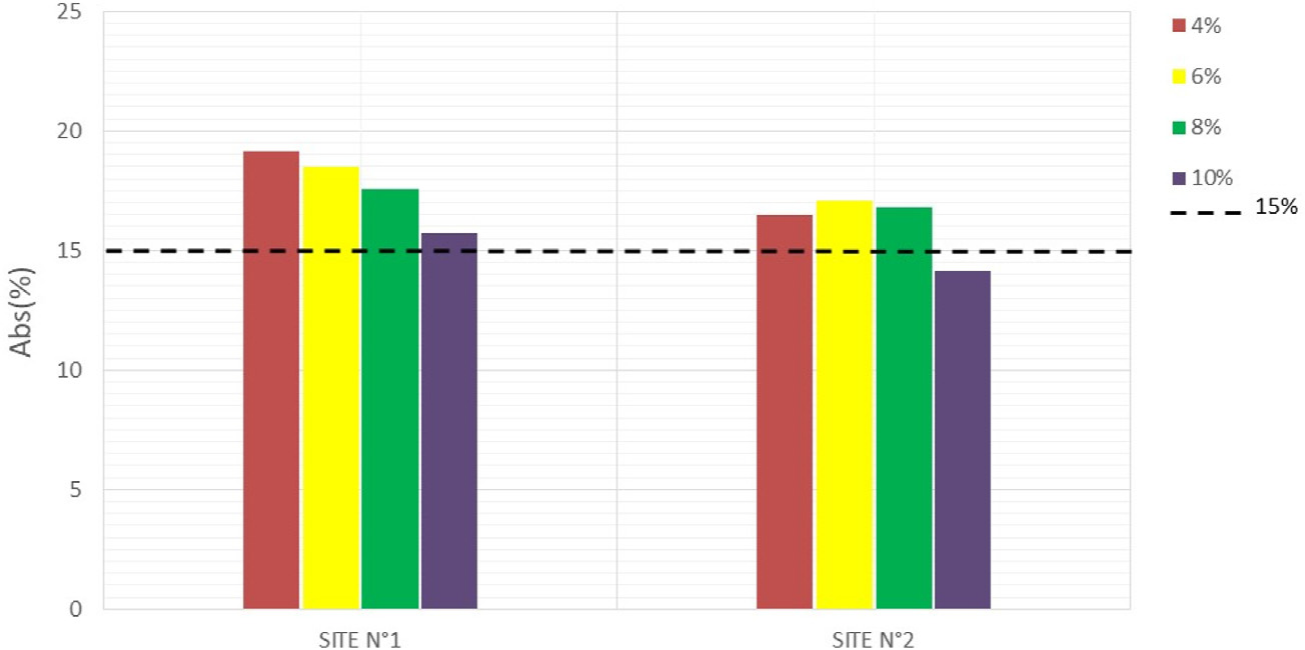
14- days water absorption

**Figure 18:**
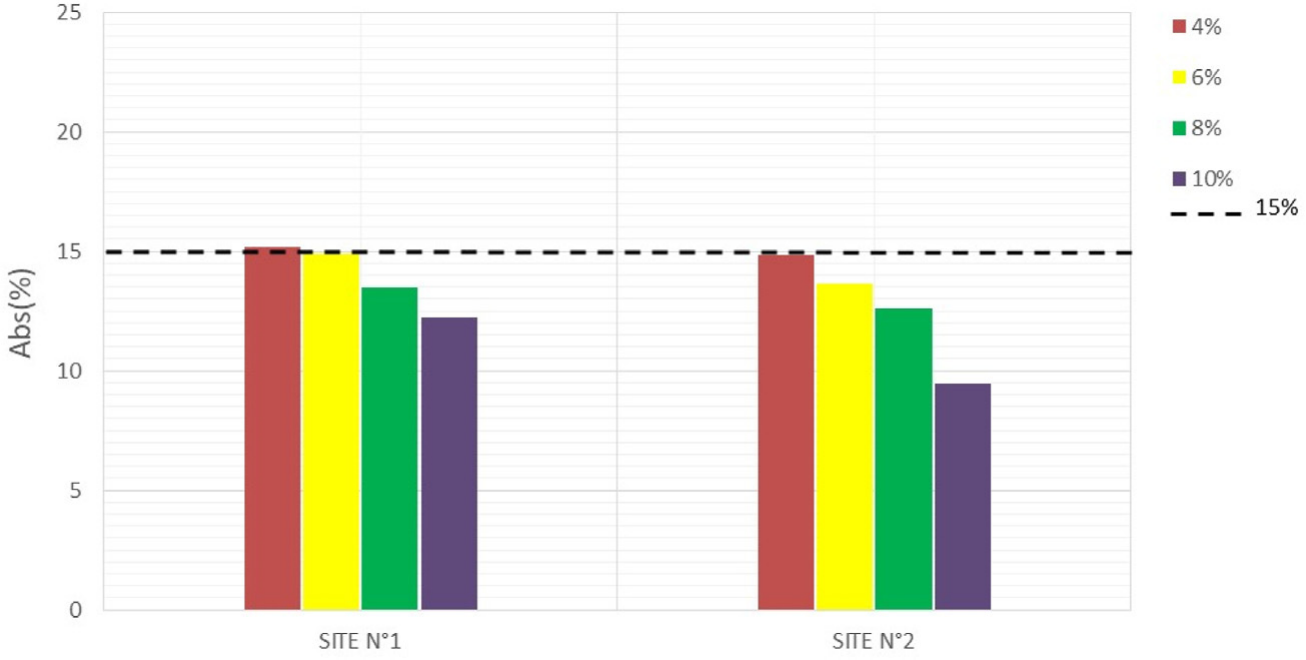
28- days water absorption

Water absorption tests on the specimens were conducted to assess the durability of the manufactured mud bricks. The less absorbent a brick is, the better it is at withstanding weathering (Kerali, 2001). The results obtained are recorded in Table 6, and Figures 16, 17, and 18 illustrate the variations in water absorption percentages.

**Table 6:** Water absorption.

SITES		Water absorption		
		7 days	14 days	28 days
**N°1**	0%	/	/	/
	4%	20,82	19,15	15,17
	6%	20,33	18,49	14,89
	8%	19,88	17,57	13,48
	10%	17,76	15,71	12,26
**N°2**	0%	/	/	/
	4%	20,15	16,50	14,85
	6%	20,07	17,11	13,65
	8%	17,05	16,84	12,62
	10%	14,92	14,12	9,46

**Figure 19:**
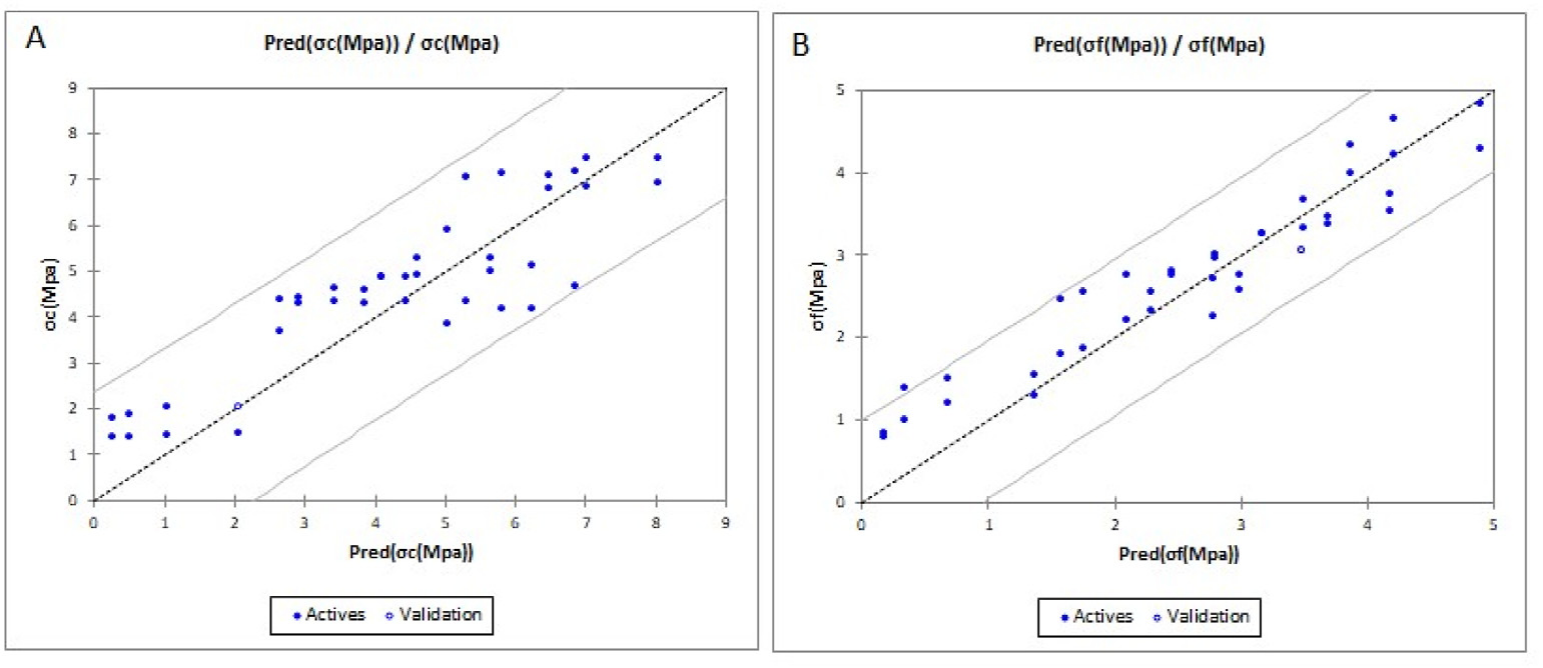
Graphs showing experimental values versus predicted values; A: first model; B: Second model.

At 0% cement, the specimens disintegrate completely after 24 h of immersion in water. But with the addition of 4% cement, the water absorption of the material decreases as the stabilization rate increases. These results show that the addition of cement makes the bricks less absorbent and more insensitive to water. Studies by Ganou Koungang et al. (2019) show that cement improves the water properties of bricks through a physicochemical reaction between the soil particles and the added cement, creating a matrix that binds the particles together. The NC_102-115 (2007) standard prescribes absorption rate values of less than 15% for the use of BECs in construction. At 28 days, the compressed and stabilized soil brick samples comply with this standard.

#### 5.4.3 Physical parameters of compressed earth bricks

Density is a parameter that is studied to determine the quality of compaction. The results obtained are usually related to the strength and type of compaction used. Other characteristics are also taken into account, such as the characteristics of the materials used and the water content during the pressing process. Tables 7 and 8 and 9, and show the results obtained at 7, 14, and 28 days.

**Table 7:** Bulk densities of the specimens at 7 days.

SITES		dimensions (cm)			mh (g)	V (cm3)	ρ (g/cm3)
		L	l	H			
N°1	0%	8,1	4	1,7	95,42	55,08	1,73
	4%	8,2	4	1,6	99,8	52,48	1,90
	6%	8,2	4	1,7	97,92	55,76	1,75
	8%	8,2	4	1,6	97,8	52,48	1,86
	10%	8,2	4	1,6	99,2	52,48	1.89
N°2	0%	8,1	4	1,6	90,5	51,84	1,74
	4%	8,2	4	1,6	99,83	52,48	1,91
	6%	8,2	4,1	1,5	98,55	50,43	1,95
	8%	8,2	4	1,6	99,96	52,48	1,91
	10%	8,2	4	1,6	99,89	52,48	1,91

**Table 8:** Bulk density of specimens at 14 days.

SITES		Dimensions (cm)			mh (g)	V (cm3)	ρ (g/cm3)
		L	l	H			
N°1	0%	8,1	4	1,5	95,42	48,6	1,96
	4%	8,2	4	1,6	99,8	52,48	1,91
	6%	8,2	4,1	1,7	97,92	57,15	1,71
	8%	8,2	4	1,6	97,8	52,48	1,86
	10%	8,2	4,1	1,6	99,2	53,79	1,84
N°2	0%	8,1	4	1,5	90,5	48,6	1,86
	4%	8,2	4	1,5	97,83	49,2	1,98
	6%	8,2	4	1,6	99,72	52,48	1,90
	8%	8,2	4,1	1,6	99,96	53,79	1,85
	10%	8,2	4,1	1,5	99,89	50,43	1,98

**Table 9:** Bulk densities of the specimens at 28 days.

SITES		Dimensions (cm)			mh (g)	V (cm3)	ρ (g/cm3)
		L	l	H			
N°1	0%	8,1	4	1,5	95,42	48,6	1,96
	4%	8,2	4	1,5	97,8	49,2	1,98
	6%	8,2	4	1,6	97,92	52,48	1,86
	8%	8,2	4	1,6	97,8	52,48	1,86
	10%	8,2	4,1	1,6	99,2	53,79	1,84
N°2	0%	8,1	4	1,5	90,5	48,6	1,86
	4%	8,2	4	1,6	97,83	52,48	1,86
	6%	8,2	4	1,6	99,72	52,48	1,90
	8%	8,2	4	1,5	97,96	49,2	1,99
	10%	8,2	4	1,5	97,89	49,2	1,98

The values that the standard NC_102-115 (2007) recommend for the BEC vary between 1.5 and 2 g/cm^3^. The samples of the study area are in this range. They present with rates of cement from 0 to 10%, values between 1.7 and 1.99 g/cm^3^. This allows us to conclude that the compaction was well executed.

These results also show that the drying time of compressed earth bricks does not have a great influence on the dimensions of the material. After the installation of these bricks, the linear and volumetric shrinkage is negligible.

### 5.5 Statistical analysis and prediction model descriptive statistics

The values obtained by the descriptive statistics (minimum, maximum, median, mean, variance, and standard deviation) of the studied data (stabilized and non-stabilized at 7, 14, 28, and 56 days) are recorded in Table 10. The Pearson correlation table (Table 11) between the different parameters studied shows a strong correlation (−0.80) between time and water uptake of bricks. This negative correlation illustrates the fact that the water absorption evolves inversely to time, the more time passes, the more the stabilized earth brick tends to absorb less water. There is a strong positive correlation between cement content and the mechanical properties of bricks. This correlation confirms previous studies which state that the addition of cement during the manufacture of compressed earth bricks increases their mechanical performance. A negative correlation is also observed between the cement content and the water absorption rate. This correlation reflects the reduction of water absorption with the addition of cement. In addition, there is also a negative correlation between mechanical parameters (compressive strength and flexural strength) and water absorption. The mechanical parameters tend to be better when water absorption rates are lower.

**Table 10:** Descriptive statistics of CEB parameters.

Statistics	t(d)	cement (%)	σc (Mpa)	σf (Mpa)	Abs (%)	σc (Mpa)
No. of observations	40,00	40,00	40,00	40,00	40,00	40,00
Minimum	7,00	0,00	1,38	0,79	9,46	1,38
Maximum	56,00	10,00	7,47	4,83	20,82	7,47
Median	21,00	6,00	4,62	2,75	16,67	4,62
Average	26,25	5,60	4,59	2,75	16,37	4,59
Variance (n-1)	361,22	12,14	3,38	1,12	8,72	3,38
Std. Deviation (n-1)	19,01	3,48	1,84	1,06	2,95	1,84

**Table 11:** Correlation matrix of CEB parameters (Pearson).

Variables	t(d)	Cement (%)	σc (Mpa)	σf (Mpa)	Abs (%)
**t(d)**	**1,00**	0,00	0,34	0,36	**-0,80**
**Cement (%)**	0,00	**1,00**	**0,62**	**0,86**	**-0,48**
**σc(Mpa)**	0,34	**0,62**	**1,00**	**0,74**	**-0,47**
**σf(Mpa)**	0,36	**0,86**	**0,74**	**1,00**	**-0,73**
**Abs (%)**	**-0,80**	**-0,48**	**-0,47**	**-0,73**	**1,00**

#### 5.5.1 Data modeling

The correlation test allowed the identification of parameters that are related to each other allowing the establishment of some prediction models of the mechanical parameters of the bricks. The models generated by the regression analysis are:

**First model:** modeling of compressive strength as a function of cement content and time. This model is given by Eq. (1) with the corresponding coefficients

(1) σc(Mpa) = 0,50832∗cement+0,08273∗t(d)

R = 0.87; R^2^ = 0.75

**Second model:** Modeling of flexural strength as a function of cement content and time. This model is given by Eq. (2) with the corresponding coefficients

(2) σf(Mpa) = 0,30394∗cement+0,05042∗t(d)

R = 0.96; R^2^ = 0.93

The correlation coefficient (R) and the coefficient of determination (R^2^) of these models indicate that the values predicted by the models are very close to the measured values. To validate the models developed, measured laboratory data independent of the mathematical model constructed, i.e. whose variables were not used to generate these prediction models, were employed. The residuals obtained between the measured and predicted values presented in Tables 12 and 13 and illustrated in Figure 19, show that the difference between the model and reality is not very important. The cross-validation table of the model presents the measured data and the corresponding predicted values.

**Table 12:** Cross-validation table of the first model.

Observation	σc (Mpa)	Pred (σc (Mpa))	Residual
**Obs7**	3,6950	2,6124	1,0826
**Obs12**	4,4150	3,1915	1,2235
**Obs13**	4,8650	4,2081	0,6569
**Obs14**	7,0500	5,2247	1,8253
**Obs21**	2,0500	2,3164	-0,2664
**Obs23**	5,2700	5,3663	-0,0963
**Obs25**	7,4500	7,3995	0,0505
**Obs28**	4,9050	5,3663	-0,4613

**Table 13:** Cross-validation table of the second model.

Observation	σf (Mpa)	Pred (σf (Mpa))	Residual
**Obs3**	2,3300	2,1766	0,1534
**Obs5**	3,3750	3,3924	-0,0174
**Obs15**	4,3330	3,7453	0,5877
**Obs18**	2,7500	2,5295	0,2205
**Obs20**	4,0000	3,7453	0,2547
**Obs22**	2,2080	2,6275	-0,4195
**Obs25**	4,6600	4,4512	0,2088
**Obs27**	2,7500	2,6275	0,1225

Using the models thus obtained, the mechanical properties of CEBs using the studied soils can be predicted on the basis of the cement content and the curing time.

## 6 Conclusion

The present work consisted of the identification, characterization, and classification of soils in the locality of Nkoulou, in the Central Cameroon region, for building and road construction. To do this, several investigations were undertaken. It consisted in carrying out a field campaign with the task of reconnaissance, taking samples, and recording field data. Subsequently, laboratory analyses were carried out to determine the physical, mechanical, chemical, and mineralogical parameters of these soil samples. The last step consisted of the interpretation of the results, the analysis of the statistical correlations between them, and the determination of prediction equations with the most correlated data.

The field investigations concerned 02 sites (site n°1 and site n°2). Each of these sites presented 04 lithological layers including arable soil on the surface, followed by a layer of sandy-clay texture comprising nodules of laterite armor (Site n°1) or formed of nodules with variable granulometry (site n°2). These layers overhang isalterites that rest on the parent rock. A total of 08 samples were taken, of which 04 per site.

Chemical analysis of the base oxides by XRF showed that the soils in the study area were ferric-dominated laterites. The Fe_2_ O_3_ content varied between 20.01 and 45.31%. SiO_2_ was present in moderate proportions (between 20.82% and 38.73%). The content of Al_2_ O_3_ was low to moderate (between 17.48 and 24.15%) and the other oxides (Cao, MgO, SO_3_, K_2_O, Na_2_O, P_2_O_5_) were weakly represented with contents lower than 1.00%. The loss on ignition varied around 11%. The proportion of organic matter in the studied soils varied between 3.54% and 6.44%, which would have a negligible impact on the geotechnical use of the material. Mineralogical analysis by XRD showed that the laterites consisted mainly of quartz, illite, hematite, kaolinite, goethite, gibbsite, muscovite, and magnesite, accompanied by other minerals including montmorillonite found in traces. The identified minerals offered the material plastic properties (illite), swelling (montmorillonite), and workability (Kaolinite) among others.

Regarding the physical test results, the analysis of the natural water content showed values ranging from 8.1% to 15.4%, with an average of 11.73%. The relatively high values of water content (LML 02 and LML 04) were because the sampling campaigns were carried out in April, the month of the beginning of the rainy season. The results of the particle size distribution analysis reported that the soils studied were mainly composed of gravel followed by sand, except for LML 04 and LML 07 which contained more clay particles. These soils were classified as Group B fine-grained soils according to the French road grading manual (GTR). A strong correlation was found between the natural water content (W) with the clay content (A%) and the silt content (L%). This coefficient was 0.75 between natural water content and clay, and 0.73 between natural water content and silt. This reflected the fact that increasing clay and silt content tends to increase the natural water content of the soils. The average values of specific gravity were 2.51 and 2.48 respectively, for the soils of site N°1 and site N°2. Methylene blue showed a good correlation with silt content (0.87) and clay content (0.61), showing that silt content had a more significant effect on the methylene blue value. Observation of the plasticity chart data indicated that the majority of the lateritic soils in the study area were in the low plastic clay to high plastic silt zone, except for sample LML 03 which was in the high plastic clay zone. A good correlation was established between the liquidity limit and the contents of CaO and Fe_2_O_3_. This result indicated that these elements had mainly an effect on the plasticity and therefore they would be the major oxides of the clays present in the studied soils. The Proctor curves of the studied soils presented average curvatures according to CRATERRE; they could thus be used as well in the manufacture of BTC as in the realization of road works in layers of fill. According to the results of the CBR test, the soils studied were class S4 soils that could be used in the top layer of rail and the foundation layer for T1 traffic (DEGN, 1987). The CBR index of the studied soil tended to decrease when the percentage of silt increased, a remark deduced from the correlation (−0.69) between the CBR index (ICBR) and the silt content. On the other hand, the good correlation (0.73) between the maximum dry density and the bearing capacity index (ICBR) indicated that the greater the maximum dry density, the greater the bearing capacity of the soil. The Proctor curves of the studied soils presented average curvatures according to CRATERRE; they could thus be used as well in the manufacture of BTC as in the realization of road works in layers of the embankment.

Regarding the mechanical tests, the results showed that at 0% stabilization with cement, the compressive strength hardly changed over time, while with the addition of cement, a significant increase in strength was observed between 7 and 14 days. Moreover, it was noted that the mechanical resistances remained constant after a period of 14–28 days. This relative constancy showed that the compressed earth bricks reached their optimal strength at this period. The unstabilized CTBs disintegrated when immersed in water for 24 h while the presence of cement maintained the cohesion of the immersed bricks. All the CTP tested met the NC_102-115 (2007) standards for water absorption. The regression analysis allowed the generation of 02 mathematical models for the prediction of mechanical resistance:

- Modelling of compressive strength as a function of cement content and time **σc(Mpa) = 0,50832∗cement+0,08273∗t(d) (1) with** R = 0.87; R^2^ = 0.75;
- Modeling of flexural strength as a function of cement content and time **σf(Mpa) = 0,30394∗cement+0,05042∗t(d) (2) with** R = 0.96; R^2^ = 0.93.

## Declarations

### Author contribution statement

Tchedele Langollo Yannick: Conceived and designed the experiments; Performed the experiments; Analyzed and interpreted the data; Contributed reagents, materials, analysis tools or data; Wrote the paper.

Oumar Ali Taïga; Taypondou Darman; Mambou Ngueyep Luc Leroy; Mache Jacques Richard: Conceived and designed the experiments; Analyzed and interpreted the data; Contributed reagents, materials, analysis tools or data; Wrote the paper.

Leke Fekwe Kom Michel Ivan: Performed the experiments; Analyzed and interpreted the data; Contributed reagents, materials, analysis tools or data.

Liyong Luc Arnold: Analyzed and interpreted the data; Contributed reagents, materials, analysis tools or data; Wrote the paper.

### Funding statement

This research did not receive any specific grant from funding agencies in the public, commercial, or not-for-profit sectors.

### Data availability statement

Data will be made available on request.

### Competing interest statement

The authors declare no conflict of interest.

### Additional information

No additional information is available for this paper.
